# Supplementary material for: Barriers and enablers to improving integrated primary healthcare for non-communicable diseases and mental health conditions in Ethiopia: a mixed methods study
Source: BMC Prim Care. 2024 Jun 11;25:211. doi: 10.1186/s12875-024-02458-6 (PMC11167879; doi:10.1186/s12875-024-02458-6)
Supplement: Supplementary file 1 — Supplementary Material 1. [file 12875_2024_2458_MOESM1_ESM.docx]

# Topic guide: Healthcare workers, managers and administrators (NCD/mental health study)

Before we start the interview, I would like to ask you a few details about yourself:

| 1 | Age | ___ ___ years | |
| --- | --- | --- | --- |
| 2 | Gender | Male |  |
|  |  | Female |  |
| 3 | Qualification | Diploma  Specify: |  |
|  |  | BSc  Specify: |  |
|  |  | Masters  Specify: |  |
|  |  | Other qualification  Specify: |  |
| 4 | Number of years of work experience | ____ ____ |  |
| 5 | Place of work | Butajira hospital |  |
|  |  | Buee hospital |  |
|  |  | Other hospital  Specify: |  |
|  |  | Health centre  Specify: |  |
| 6 | Position | Health worker (general OPD) |  |
|  |  | Health worker (NCD/mental health OPD) |  |
|  |  | Health centre manager |  |
|  |  | HMIS focal person |  |
|  |  | NCD/NTD/mental health focal person |  |
|  |  | Other (specify) |  |

As I explained, we are interested to hear about your experience providing care for people with NCDs, mental health or substance use disorders and how it could be improved. What you tell us is confidential so please feel comfortable to speak frankly with us.

1. **[Training and use of Ethiopia PCC guidelines]**

What experience do you have providing care for people with NCDs? Probe for each separately: Diabetes? Hypertension? Heart disease (cardiovascular disease)? Chronic respiratory disease? (e.g. asthma).

What experience do you have providing care for people with depression?

What experience do you have providing care for people with substance use problems? E.g. alcohol-related problems? Khat-related problems?

*[in health facility-based health workers]*

How did you find the training on treatment for people with NCDs, depression or substance use problems in the Ethiopia PCC guidelines? How helpful was the training? How confident were you to deliver care after being trained? What else did you need so that you would be confident to deliver the service for people with these types of problem?

*[for district/zonal administrators]*

What do you think about the way training is being provided for the new Ethiopia PCC guidelines? To what extent do you think health workers will feel confident to follow the guidelines after the training? What else is needed?

What barriers are there to delivering care for people with NCDs in health centres? What about depression? What about substance use problems?

*[for all]*

How useful are the new Ethiopia PCC guidelines for these problems (NCDs, mental health and substance use problems)? Is there anything you think needs to be changed about the guidelines? If so, what and why?

*[healthcare workers only]*

How much do you use the guidelines? What gets in the way of you using the guidelines? What would help you to use the guidelines more frequently?

1. **[Needs of people with NCDs, mental health and substance use problems]**

What do you think people with NCDs need when they come to the health facility? What do you think patients want from the service? Anything else? How satisfied do you think they are with the service?

In your view, what could improve the service at this health facility for people with NCDs?

What about in the community? Are there actions that need to be taken in the community to help people with NCDs? Tell me about these?

How about people with depression? What do you think they want from the service here? Anything else? How satisfied do you think they are with the care?

In your view, what could improve the service at this health facility for people with depression?

What about in the community? Are there things that need to be done in the community to help people with depression?

How about people with substance use problems? What do you think they want from the service here? Anything else? How satisfied do you think they are with the care?

In your view, what could improve the service at this health facility for people with substance use problems?

What about in the community? Are there actions that need to be taken in the community to help people with substance use problems? Tell me about these?

Many people have more than one problem at the same time e.g. they could have diabetes and hypertension, or khat use and hypertension, or asthma and depression. How well do you think co-morbid conditions are detected in this health facility (i.e. when there are 2 or more health problems at the same time)? How often do you screen for other (related) problems when a person is diagnosed as having an NCD or depression or substance use problem? What would get in the way of screening for these problems routinely? What do you think about that?

1. **[care pathways and HMIS for people with NCDs, mental health and substance use problems]**

*[detection]*

How do people find out that they have an NCD (e.g. hypertension, diabetes, heart problem (cardiovascular disease), chronic respiratory problem), depression or substance use? [probe separately]

How much delay is there for people with NCDs to come to the health facility? Why is there a delay?

When people come to the health facility, how are NCDs detected? In your view, how good is the detection of NCDs in this facility? How could it be improved?

*[engagement on an evidence-based care pathway]*

When an NCD, depression or substance use problem is detected at the health facility, what happens next? How does the healthcare worker decide what care to give?

What monitoring is in place to check whether or not the person is getting the correct care? If this could be monitored, what benefits could that bring? What could be the disadvantages?

*[adherence]*

How is patient adherence to treatment monitored? How could this be improved? How could patients be supported to take their treatment as prescribed?

How about changes in lifestyle e.g. diet, exercise, stopping substance use? How do you check what patients are doing? How could patients be supported to make these changes?

*[retention]*

How many people drop out of care? How do you know whether people have dropped out of care? What systems are present to detect drop-out? What systems are present to do something about drop-out? What do you think could be improved? What do you think needs to happen when someone drops out of care?

*[treatment-to-target]*

How do you assess whether a person is responding properly to the treatment? How do you know when they are treated adequately? If you think about all the people you are treating at the moment, do you know how many of them are responding to treatment as expected? How is response to treatment monitored? Could this be improved?

*[electronic data]*

What do you think about collecting electronic information to know about what care people are getting, their drop-out from care and how well they are responding to treatment? How could this work in practice? What could be the benefits? How about the disadvantages?

1. **[possible health system strengthening interventions]**
2. [Clinical communication skills/handling emotions]

How important do you think it is to ask about patients’ emotional state? Or their social problems?

How comfortable are you asking patients about their emotional state? What makes you uncomfortable? How about asking patients about their social problems? What gets in the way of asking about these types of patient concerns?

What do you think of the idea of having training and support to ask about emotional problems in patients?

1. [Quality improvement]

How could quality of care be improved in this facility? How often is the quality of care discussed? What do you think about the idea of meeting regularly to look at the quality of care? How could this be used for learning? How could this avoid blaming individuals?

1. [Self management/person-centred care]

What ways are there to improve the awareness that patients have about their health problems? How could this be made into a routine activity?

How do you think patients could become more active in handling their health problems? E.g. being motivated to make positive changes in their lifestyle?

How much do you ask patients about their preferences for treatment? What do you think about this idea? What would be the barriers to involving patients in making decisions about treatment? What could be the advantages?

1. Any other comments on how care for people with NCDs, mental health and substance use problems could be improved?

Thank you very much for your time and for sharing your ideas on these topics.
